# An updated systematic review and meta-analysis on *Mycobacterium tuberculosis* antibiotic resistance in Iran (2013-2020)

**DOI:** 10.22038/IJBMS.2021.48628.11161

**Published:** 2021-04

**Authors:** Farzad Khademi, Amirhossein Sahebkar

**Affiliations:** 1 Department of Microbiology, School of Medicine, Ardabil University of Medical Sciences, Ardabil, Iran; 2 Halal Research Center of IRI, FDA, Tehran, Iran; 3 Neurogenic Inflammation Research Center, Mashhad University of Medical Sciences, Mashhad, Iran; 4 Biotechnology Research Center, Pharmaceutical Technology Institute, Mashhad University of Medical Sciences, Mashhad, Iran

**Keywords:** Antibiotic, Iran, Meta-analysis, Mycobacterium tuberculosis, Resistance

## Abstract

This updated systematic review and meta-analysis follows two aims: 1) to assess *Mycobacterium tuberculosis (M. tuberculosis)* antibiotic resistance in Iran from 2013 to 2020 and, 2) to assess the trend of resistance from 1999 to 2020. Several national and international databases were systematically searched through MeSH extracted keywords to identify 41 published studies addressing drug-resistant *M. tuberculosis* in Iran. Meta-analysis was done based on the PRISMA protocols using Comprehensive Meta-Analysis software. The average prevalence of resistance to first- and second-line anti-TB drugs, multidrug-resistant TB (MDR-TB) and extensively drug-resistant TB (XDR-TB) in new and previously treated tuberculosis (TB) cases in Iran during 2013–2020 were as follows: isoniazid 6.9%, rifampin 7.9%, ethambutol 5.7%, pyrazinamide 20.4%, *para*-aminosalicylic acid 4.6%, capreomycin 1.7%, cycloserine 1.8%, ethionamide 11.3%, ofloxacin 1.5%, kanamycin 3.8%, amikacin 2.2%, MDR-TB 6.3% and XDR-TB 0.9%. Based on the presented data, *M. tuberculosis* resistance to first- and second-line anti-TB drugs, as well as MDR-TB, was low during 2013–2020 in Iran. Furthermore, there was a declining trend in TB drug resistance from 1999 to 2020. Hence, to maintain the current decreasing trend and to control and eliminate TB infection in Iran, continuous monitoring of resistance patterns is recommended.

## Introduction

On 24 March 1882, Dr Robert Koch discovered a rod-shaped, aerobic bacterium called *Mycobacterium tuberculosis* (*M. tuberculosis*). It is the causative agent for tuberculosis (TB) which is one of the ten most common causes of death worldwide. The latent form of tubercle bacilli has been detected in 1.7 billion people around the world (1-3). Between 5 and 10% of these people will eventually develop the active form of the disease (pulmonary or extrapulmonary) in their lifetime (4, 5). TB is an air-borne and communicable disease and humans are the only natural reservoir of the infection (6). According to the latest report of the World Health Organization (WHO) in 2018, TB remained a global health problem with 10 million infected people (57% adult men, 32% adult women, and 11% children), 1.2 million deaths in HIV-negative and 251,000 deaths in HIV-positive patients (1). In 2014, WHO announced “the End TB Strategy” which aimed to reduce TB-associated deaths (by 90%) and incidence rate (by 80%) and to end the global TB epidemic between 2016 and 2035 (1). To achieve these purposes, developing novel diagnostic tests for rapid detection, use of effective drugs or new treatments, and appropriate vaccination are needed (1). Since 1921, the bacilli Calmette-Guérin (BCG) live attenuated vaccine has been the only approved vaccine for TB prevention in newborns and children. It has variable protection against adult pulmonary TB (0–80%) while it is unable to prevent the reactivation of latent TB (7, 8). Treatment with first-line drugs i. e., isoniazid, rifampin, ethambutol, and pyrazinamide is recommended for a six-month period in drug-susceptible TB as the mortality rate is high without treatment (1). The success rate for first-line drugs is >85%, however, the emergence of drug-resistant *M. tuberculosis*, particularly multidrug-resistant TB (MDR-TB defined as resistance to isoniazid and rifampin) has led to a decrease in treatment success to 56%, an increase in treatment duration and costs, and administration of more toxic second-line drugs (1). TB incidence in 2018 in Iran, a country with a population of 82 million located in the Middle East, showed a decreasing trend (11, 000; 14 cases per 100,000 population), however, it has shared geographical borders with high incidence countries such as Pakistan (6% in 2018) (1). Hence, continuous surveillance on TB epidemiology especially drug resistance status is of high necessity for disease control. *M. tuberculosis* antibiotic resistance pattern has previously been studied between March 1999 and May 2013 in Iran (9). Nevertheless, as the prevalence of TB antibiotic resistance is changing over time, we have performed the current systematic review and meta-analysis from 2013 to 2020 to update the evidence in Iran.

## Methods

***Search strategies and data sources***

The current systematic review and meta-analysis was conducted based on the PRISMA (Preferred Reporting Items for Systematic Reviews and Meta-Analysis) guidelines (10). The computer-assisted systematic search was done in national and international databases including Scientific Information Database (SID) (www.sid.ir), Magiran (www.Magiran.com), ISI Web of Knowledge, PubMed, and Scopus. Each cross-sectional study published between May 2013 and March 30, 2020, in English or Persian languages on *M. tuberculosis* antibiotic resistance in Iran was investigated. *M. tuberculosis*, antibiotic resistance, and Iran were the main medical subject heading (MeSH) extracted keywords used for searching. The reference lists of all included articles were further assessed to find any missed relevant studies.

**Data selection criteria and quality assessment**

The titles, abstracts, and full texts of collected articles were further evaluated in detail to select eligible studies based on our inclusion and exclusion criteria. In addition, the Joanna Briggs Institute (JBI) critical appraisal checklist was selected to assess the quality of each included article. Eligible studies showed high quality (>5 scores), medium quality (4-5 scores), or low quality (<4 scores) (11). Original articles assessing the prevalence of *M. tuberculosis* antibiotic resistance with the following features were included in the study: (1) published in English or Persian, 2) limited to Iran, 3) clinical strains of *M. tuberculosis* isolated from new or previously treated cases, 4) studies evaluating monoresistance (which is defined as resistance to only 1 first- or second-line anti-TB drugs), MDR and XDR (extensively drug-resistant) status and 5) full-text availability. The following studies were excluded from analysis: 1) studies reporting drug resistance patterns in nontuberculous mycobacteria (NTM), 2) studies that only focused on drug resistance mechanisms, 3) studies that solely evaluated any drug resistance status (which is defined as resistance to any drug regardless of monoresistance), 4) non-original studies, 5) duplicate publications including same studies published in both English and Persian languages and two or more relevant studies conducted by the first or corresponding author in the same year and also several multicenter studies with identical information such as same authors’ name, same enrollment time and previously determined antibiotic resistance profiles, 6) studies available only in abstract form and dissertations, 7) studies without sufficient data such as study date or ambiguous information, 8) studies performed merely to evaluate the sensitivity and specificity of different antimicrobial susceptibility testing methods using previous banks of bacterial isolates or clinical isolates of *M. tuberculosis* with known drug resistance profiles, 9) studies on *M. tuberculosis* other than antibiotic resistance such as epidemiological patterns or treatment of the disease, and 10) multicenter studies without sufficient and separate data for each province.

**Data extraction**

Extracted information from eligible studies included in the meta-analysis is shown in Table 1. Important data were as follows: first author’s surname, publication date, region of study, study enrollment date, number of tested isolates, methods used for assessing bacterial antibiotic susceptibility, and prevalence of *M. tuberculosis* resistance to first- and second-line drugs.

**Data analysis**

Meta-analyses were conducted using the Comprehensive Meta-Analysis (CMA) (Biostat, Englewood, NJ) software package, and antimicrobial resistance trends were determined by the Graphpad Prism software package. Important analyzed parameters included the rates of *M. tuberculosis* antibiotic resistance and heterogeneity and publication bias among studies. The pooled estimates of the resistance prevalence for each drug were reported as a percentage and 95% confidence intervals (CIs) using a random-effects model (heterogeneity ≥25%;) or fixed-effects model (heterogeneity <25%;). The possibility of heterogeneity was evaluated by *I*² statistic and the Chi-square test with the Cochrane *Q* statistic. The existence of publication bias was assessed by Funnel plots.

## Results

**Figure 1:**
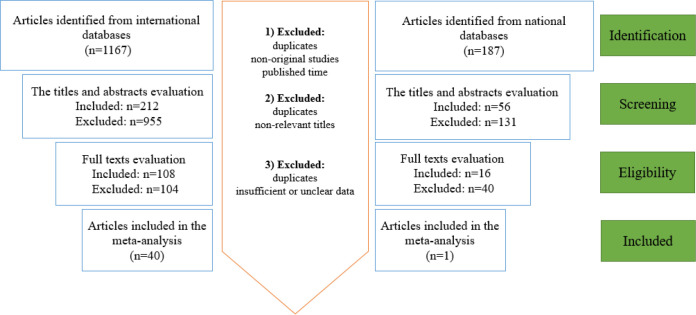
Summary of the literature search and study selection in the meta-analysis

As shown in Figure 1, a total of 1,354 articles in Persian and English languages were collected during early literature search. At the end of the screening, 41 eligible articles were included in the meta-analysis based on the predefined inclusion and exclusion criteria. Forty-one included studies describing the prevalence of drug-resistant *M. tuberculosis* were selected from different provinces of Iran (Table 1). The most common laboratory methods that were used for assessing *M. tuberculosis* antibiotic susceptibility were agar proportion and molecular techniques such as line probe assay (LPA), real-time polymerase chain reaction (PCR), multiplex allele-specific polymerase chain reaction (MAS-PCR), and multiplex PCR.

**Figure 2:**
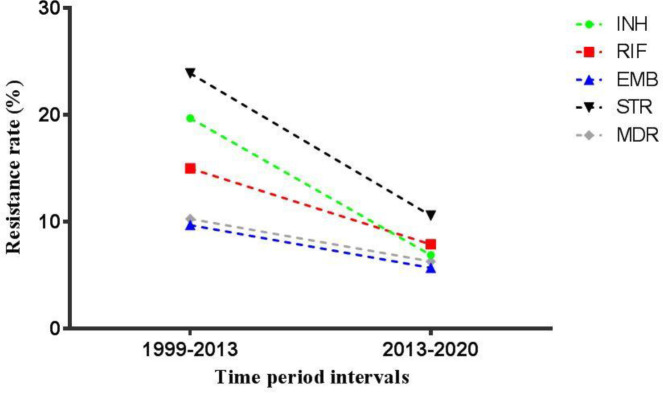
Antimicrobial resistance trends of *Mycobacterium tuberculosis* strains to isoniazid, rifampin, ethambutol, streptomycin, as well as MDR-TB, among new and previously treated cases in Iran from 1999 to 2020

The total prevalence of isoniazid-resistant* M. tuberculosis* among new and previously treated cases in Iran was 6.9% (95% *CI*: 5.5-8.6) between 2013 and 2020, which was estimated using a random-effects model due to existence of significant heterogeneity (*I*^2^ = 92.1%; *Q* = 1101.5; *P* = 0.00). As shown in Table 2, the highest prevalence of isoniazid-resistant *M. tuberculosis* strains in different provinces of Iran was found in Fars (16.7%) and the lowest rates were seen in Semnan (0%), Yazd (0%), and Zanjan (0%). Additionally, Figure 2 demonstrates a decreasing trend in the incidence of isoniazid-resistant strains from 2013 to 2020 (6.9%) in comparison with 1999-2013 (19.7%) in Iran. According to the random-effects model (*I*^2^=83.7%; *Q*=516.4; *P*=0.00), the prevalence of rifampin-resistant *M. tuberculosis* strains among new and previously treated cases in Iran was 7.9% (95% *CI*: 6.6-9.5) between 2013 and 2020 which was lower than the previous report from 1999 to 2013 (15%). *M. tuberculosis* strains isolated from Fars (16.1%), Semnan (0%), and Zanjan (0%) showed the highest and lowest rates of resistance to rifampin, respectively. Among new and previously treated cases between 2013 and 2020, the mean resistance to ethambutol in Iran was 5.7% (95% *CI*: 4-8). The rate of resistance was highest in Kermanshah (14.2%) and lowest in Golestan (0%). The overall prevalence of ethambutol-resistant* M. tuberculosis *strains in Iran was calculated using the random-effects model (*I*^2^ = 86%; *Q* = 208.3; *P* = 0.00). As presented in Figure 2, ethambutol-resistant strains also showed a decreasing pattern in Iran from 1999 to 2020 (9.7% to 5.7%). Frequency of *M. tuberculosis *resistance to pyrazinamide calculated using the fixed-effects model was 20.4% (95% *CI*: 15.7-26.2;* I*^2^ = 13.2%; *Q* = 11.5; *P* = 0.31). The highest resistance rate was found in Hamadan (40%) and the lowest rate was seen in Razavi Khorasan (0%). 

**Figure 3:**
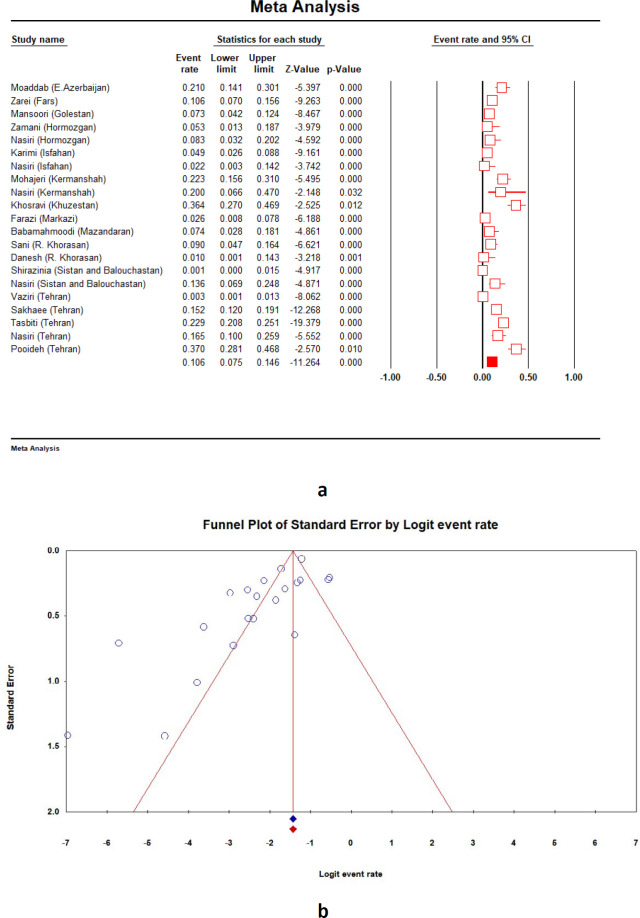
Forest plot (a) and funnel plot (b) showing streptomycin-resistant *Mycobacterium tuberculosis* prevalence among new and previously treated cases between 2013 and 2020 in Iran

Second-line anti-TB drugs (groups A-D) used for MDR treatment include 1) group A: fluoroquinolones (ofloxacin, levofloxacin, moxifloxacin, and ciprofloxacin), 2) group B: injectable drugs (aminoglycosides (kanamycin, amikacin, and streptomycin) and cyclic peptides (capreomycin)), 3) group C: ethionamide/prothionamide, cycloserine/terizidone, linezolid and clofazimine, and 4) group D: D2 (bedaquiline and delamanid) and D3 ( *para*-aminosalicylic acid, imipenem-cilastatin, meropenem, amoxicillin/clavulanate and thioacetazone). The prevalence of *M. tuberculosis *resistance to these drugs which are mainly used in treatment of MDR-TB was not completely evaluated in the previous study conducted from 1999 to 2013 in Iran. Figure 3 shows the forest plot and funnel plot of streptomycin-resistant *M. tuberculosis *strains for new and previously treated cases between 2013 and 2020 in Iran. The prevalence of *M. tuberculosis* resistance to streptomycin was estimated at 10.6% (95% *CI*: 7.5-14.6;* I*^2^ = 89.6%; *Q* = 192.8; *P* = 0.00). The maximum and minimum resistance rates were observed in Khuzestan (36.3%) and Sistan and Balouchastan (1.4%), respectively. Similar to first-line anti-TB drugs, the trend of *M. tuberculosis *resistance to streptomycin decreased between 1999 and 2020 (23.9% to 10.6%) (Figure 2). Frequency of *M. tuberculosis *resistance to other drugs during 2013 to 2020 were as follows: *para*-aminosalicylic acid 4.6% (95% *CI*: 0.3-45.1; *I*^2^ = 98.4%; *Q* = 66.5; *P* = 0.00), capreomycin 1.7% (95% *CI*: 0.5-5.4; *I*^2^ = 89.3%; *Q* = 9.3; *P* = 0.00), cycloserine 1.8% (95% *CI*: 0.5-5.9; *I*^2^ = 79.4%; *Q* = 4.8; *P* = 0.02), ethionamide 11.3% (95% *CI*: 0.6-72.9; *I*^2^ = 99.1%; *Q* = 224.5; *P* = 0.00), ofloxacin 1.5% (95% *CI*: 0.3-6.1; *I*^2^ = 91.7%; *Q* = 12.0; *P* = 0.00), kanamycin 3.8% (95% *CI*: 0.6-20; *I*^2^ = 96.7%; *Q* = 91.4; *P* = 0.00), and amikacin 2.2% (95% *CI*: 0.4-10.9; *I*^2^ = 85.8%; *Q* = 7.0; *P* = 0.00).

Additionally, the pooled estimate of MDR- and XDR-TB in Iran were 6.3% (95% *CI*: 5-8; *I*^2^ = 86.7%; *Q* = 446.3; *P* = 0.00) and 0.9% (95% *CI*: 0.4-2.2; *I*^2^ = 78.3%; *Q* = 13.8; *P* = 0.00), respectively. Similar to first-line anti-TB drugs, the rate of MDR *M. tuberculosis* in Iran between 1999 and 2020 showed a downward trend (10.3% to 6.3%). The prevalence of MDR strains was highest in Qazvin (20%) and lowest in Hamadan, Kurdistan, Semnan, and Yazd (0%).

**Table 1:** Extracted information from eligible studies included in the meta-analysis during 2013-2020

Author (Ref)	Published date	Province	Enrollment date	Strain (n)	DST	Antibiotic resistance (n)																
						First-line drugs					Second-line drugs											
						INH	RIF	EMB	PZA	STR		PAS	CAP	CYC	ETO	OFX	KAN	AMK	MDR		XDR	
Moradi et al (11)	2017	Ardabil	2014-2015	9	Agar proportional	1	0	ND	1	ND		ND	ND	ND	ND	ND	ND	ND	ND		ND	
Atashi et al (12)	2017	Ardabil	2014	14	Agar proportional	2	0	ND	ND	ND		ND	ND	ND	ND	ND	ND	ND	ND	ND	
Sahebi et al (13)	2016	Ardabil	2014	9	Molecular	3	1	ND	ND	ND		ND	ND	ND	ND	ND	ND	ND	ND	ND	
Sahebi et al (14)	2015	Ardabil	2011-2013	26	Molecular	3	4	ND	ND	ND		ND	ND	ND	ND	ND	ND	ND	ND	ND	
Velayati et al (15)	2014	Ardabil	2010-2011	65	Molecular	2	4	ND	ND	ND		ND	ND	ND	ND	ND	ND	ND	4	ND	
Moradi et al (11)	2017	East Azerbaijan	2014-2015	21	Agar proportional	2	2	ND	6	ND		ND	ND	ND	ND	ND	ND	ND	ND	ND	
Atashi et al (12)	2017	East Azerbaijan	2014	28	Agar proportional	0	1	ND	ND	ND		ND	ND	ND	ND	ND	ND	ND	ND	ND	
Sahebi et al (13)	2016	East Azerbaijan	2014	28	Molecular	1	4	ND	ND	ND		ND	ND	ND	ND	ND	ND	ND	ND	ND	
Rashedi et al (16)	2015	East Azerbaijan	2012-2014	48	Agar proportional	2	4	2	ND	ND		ND	ND	ND	ND	ND	ND	ND	1	ND	
Sahebi et al (14)	2015	East Azerbaijan	2011-2013	87	Molecular	3	15	ND	ND	ND		ND	ND	ND	ND	ND	ND	ND	ND	ND	
Moaddab et al (17)	2015	East Azerbaijan	2009-2012	100	Agar proportional	4	5	1	18	21		ND	ND	ND	ND	ND	ND	ND	21	ND	
Amini et al (18)	2019	Fars	2015-2017	19	Agar proportional	3	1	0	ND	ND		ND	ND	ND	ND	ND	ND	ND	1	ND	
Zarei et al (19)	2017	Fars	2012-2014	199	Agar proportional	38	30	16	ND	21		ND	ND	ND	ND	ND	ND	ND	22	ND	
Honarvar et al (20)	2015	Fars	2012-2013	92	Agar proportional	16	19	ND	ND	ND		ND	ND	ND	ND	ND	ND	ND	24	ND	
Motamedifar et al (21)	2015	Fars	2004-2013	59	Agar proportional	ND	ND	ND	ND	ND		ND	ND	ND	ND	ND	ND	ND	6	ND	
Velayati et al (15)	2014	Fars	2010-2011	40	Molecular	2	5	ND	ND	ND		ND	ND	ND	ND	ND	ND	ND	5	ND	
Mansoori et al (22)	2018	Golestan	2014-2015	164	Agar proportional	3	0	0	ND	12		ND	ND	ND	ND	ND	ND	ND	ND	ND	
Velayati et al (15)	2014	Golestan	2010-2011	47	Molecular	3	2	ND	ND	ND		ND	ND	ND	ND	ND	ND	ND	2	ND	
Velayati et al (15)	2014	Guilan	2010-2011	39	Molecular	1	2	ND	ND	ND		ND	ND	ND	ND	ND	ND	ND	3	ND	
Moradi et al (11)	2017	Hamadan	2014-2015	5	Agar proportional	0	0	ND	2	ND		ND	ND	ND	ND	ND	ND	ND	ND	ND	
Atashi et al (12)	2017	Hamadan	2014	11	Agar proportional	1	0	ND	ND	ND		ND	ND	ND	ND	ND	ND	ND	ND	ND	
Velayati et al (15)	2014	Hamadan	2010-2011	21	Molecular	1	2	ND	ND	ND		ND	ND	ND	ND	ND	ND	ND	0	ND	
Zamani et al (23)	2016	Hormozgan	2012-2013	38	Agar proportional	4	ND	ND	ND	2		ND	ND	ND	ND	ND	ND	ND	3	ND	
Nasiri et al (24)	2014	Hormozgan	2010-2012	48	Agar proportional	3	2	2	ND	4		ND	ND	ND	ND	ND	ND	ND	2	ND	
Velayati et al (15)	2014	Hormozgan	2010-2011	38	Molecular	3	3	ND	ND	ND		ND	ND	ND	ND	ND	ND	ND	3	ND	
Moradi et al (11)	2017	Ilam	2014-2015	4	Agar proportional	0	0	ND	1	ND		ND	ND	ND	ND	ND	ND	ND	ND	ND	
Atashi et al (12)	2017	Ilam	2014	6	Agar proportional	1	0	ND	ND	ND		ND	ND	ND	ND	ND	ND	ND	ND	ND	
Amini et al (18)	2019	Isfahan	2015-2017	19	Agar proportional	2	1	1	ND	ND		ND	ND	ND	ND	ND	ND	ND	1	ND	
Karimi et al (25)	2017	Isfahan	2014-2015	205	Agar proportional	6	4	3	ND	10		ND	ND	ND	ND	ND	ND	ND	4	ND	
Nasr Esfahani et al (26)	2016	Isfahan	2013	32	Agar proportional	ND	ND	2	ND	ND		ND	ND	ND	ND	ND	ND	ND	ND	ND	
Nasiri et al (24)	2014	Isfahan	2010-2012	45	Agar proportional	2	2	0	ND	1		ND	ND	ND	ND	ND	ND	ND	2	ND	
Velayati et al (15)	2014	Isfahan	2010-2011	42	Molecular	5	3	ND	ND	ND		ND	ND	ND	ND	ND	ND	ND	2	ND	
Velayati et al (15)	2014	Kerman	2010-2011	24	Molecular	1	3	ND	ND	ND		ND	ND	ND	ND	ND	ND	ND	3	ND	
Mohammadi et al (27)	2018	Kermanshah	2014-2015	50	Agar proportional	ND	ND	7	ND	ND		ND	ND	ND	ND	ND	ND	ND	8	ND	
Moradi et al (11)	2017	Kermanshah	2014-2015	17	Agar proportional	1	2	ND	2	ND		ND	ND	ND	ND	ND	ND	ND	ND	ND	
Atashi et al (12)	2017	Kermanshah	2014	31	Agar proportional	1	2	ND	ND	ND		ND	ND	ND	ND	ND	ND	ND	ND	ND	
Sahebi et al (13)	2016	Kermanshah	2014	16	Molecular	1	4	ND	ND	ND		ND	ND	ND	ND	ND	ND	ND	ND	ND	
Sahebi et al (14)	2015	Kermanshah	2011-2013	51	Molecular	1	5	ND	ND	ND		ND	ND	ND	ND	ND	ND	ND	ND	ND	
Mohajeri et al (28)	2014	Kermanshah	2011-2012	112	Agar proportional	18	16	15	27	25		19	ND	4	14	ND	ND	ND	16	ND	
Nasiri et al (24)	2014	Kermanshah	2010-2012	15	Agar proportional	4	3	3	ND	3		ND	ND	ND	ND	ND	ND	ND	3	ND	
Velayati et al (15)	2014	Kermanshah	2010-2011	16	Molecular	1	2	ND	ND	ND		ND	ND	ND	ND	ND	ND	ND	1	ND	
Khosravi et al (29)	2019	Khuzestan	2016-2017	307	Agar proportional	6	10	10	ND	ND		ND	ND	ND	ND	ND	ND	ND	4	ND	
Khosravi et al (30)	2019	Khuzestan	2015-2017	37	Agar proportional	3	16	ND	ND	ND		ND	ND	ND	ND	ND	ND	ND	5	ND	
Amini et al (18)	2019	Khuzestan	2015-2017	20	Agar proportional	1	1	1	ND	ND		ND	ND	ND	ND	ND	ND	ND	1	ND	
Badie et al (31)	2016	Khuzestan	2015	64	Agar proportional	ND	ND	ND	ND	ND		ND	ND	ND	ND	ND	ND	ND	2	ND	
Khosravi et al (32)	2017	Khuzestan	2013-2014	88	Agar proportional	41	35	5	ND	32		ND	ND	ND	ND	ND	ND	ND	22	ND	
Velayati et al (15)	2014	Khuzestan	2010-2011	119	Molecular	7	3	ND	ND	ND		ND	ND	ND	ND	ND	ND	ND	6	ND	
Khosravi et al (33)	2014	Khuzestan	2010-2011	160	Molecular	18	20	ND	ND	ND		ND	ND	ND	ND	ND	ND	ND	8	ND	
Moradi et al (11)	2017	Kurdistan	2014-2015	27	Agar proportional	0	0	ND	3	ND		ND	ND	ND	ND	ND	ND	ND	ND	ND	
Atashi et al (12)	2017	Kurdistan	2014	23	Agar proportional	0	2	ND	ND	ND		ND	ND	ND	ND	ND	ND	ND	ND	ND	
Sahebi et al (13)	2016	Kurdistan	2014	12	Molecular	3	1	ND	ND	ND		ND	ND	ND	ND	ND	ND	ND	ND	ND	
Sahebi et al (14)	2015	Kurdistan	2011-2013	50	Molecular	3	3	ND	ND	ND		ND	ND	ND	ND	ND	ND	ND	ND	ND	
Velayati et al (15)	2014	Kurdistan	2010-2011	16	Molecular	2	0	ND	ND	ND		ND	ND	ND	ND	ND	ND	ND	0	ND	
Heidary et al (34)	2020	Lorestan	2014-2017	106	Agar proportional	1	1	ND	ND	ND		ND	ND	ND	ND	ND	ND	ND	4	ND	
Moradi et al (11)	2017	Lorestan	2014-2015	19	Agar proportional	0	0	ND	4	ND		ND	ND	ND	ND	ND	ND	ND	ND	ND	
Atashi et al (12)	2017	Lorestan	2014	27	Agar proportional	0	1	ND	ND	ND		ND	ND	ND	ND	ND	ND	ND	ND	ND	
Velayati et al (15)	2014	Lorestan	2010-2011	24	Molecular	0	5	ND	ND	ND		ND	ND	ND	ND	ND	ND	ND	0	ND	
Farazi et al (35)	2013	Markazi	2011-2012	115	Agar proportional	3	2	8	ND	3		ND	ND	ND	ND	ND	ND	ND	9	ND	
Velayati et al (15)	2014	Markazi	2010-2011	15	Molecular	3	3	ND	ND	ND		ND	ND	ND	ND	ND	ND	ND	2	ND	
Babamahmoodi et al (36)	2014	Mazandaran	2013	54	Molecular	2	3	ND	ND	4		ND	ND	ND	ND	ND	3	3	ND	ND	
Velayati et al (15)	2014	Mazandaran	2010-2011	26	Molecular	1	1	ND	ND	ND		ND	ND	ND	ND	ND	ND	ND	1	ND	
Atashi et al (12)	2017	Qazvin	2014	5	Agar proportional	0	0	ND	ND	ND		ND	ND	ND	ND	ND	ND	ND	ND	ND	
Velayati et al (15)	2014	Qazvin	2010-2011	10	Molecular	1	0	ND	ND	ND		ND	ND	ND	ND	ND	ND	ND	2	ND	
Velayati et al (15)	2014	Qom	2010-2011	61	Molecular	3	3	ND	ND	ND		ND	ND	ND	ND	ND	ND	ND	4	ND	
Amini et al (18)	2019	Razavi Khorasan	2015-2017	56	Agar proportional	4	5	8	ND	ND		ND	ND	ND	ND	ND	ND	ND	4	ND	
Sani et al (37)	2015	Razavi Khorasan	2012-2013	100	Agar proportional	7	7	3	ND	9		ND	ND	ND	ND	ND	ND	ND	4	ND	
Danesh et al (38)	2014	Razavi Khorasan	2011-2012	48	Agar proportional	0	0	0	0	0		ND	ND	ND	ND	ND	ND	ND	ND	ND	
Velayati et al (15)	2014	Razavi Khorasan	2010-2011	117	Molecular	10	9	ND	ND	ND		ND	ND	ND	ND	ND	ND	ND	2	ND	
Velayati et al (15)	2014	Semnan	2010-2011	21	Molecular	0	0	ND	ND	ND		ND	ND	ND	ND	ND	ND	ND	0	ND	
Hashemi Shahri et al (39)	2019	Sistan and Balouchastan	2013-2016	100	Agar proportional	2	ND	ND	ND	ND		ND	ND	ND	ND	ND	ND	ND	2	ND	
Shirazinia et al (40)	2017	Sistan and Balouchastan	2010-2013	525	Agar proportional	15	16	0	ND	0		ND	ND	ND	ND	ND	ND	ND	7	ND	
Nasiri et al (24)	2014	Sistan and Balouchastan	2010-2012	59	Agar proportional	5	3	3	ND	8		ND	ND	ND	ND	ND	ND	ND	3	ND	
Velayati et al (15)	2014	Sistan and Balouchastan	2010-2011	165	Molecular	8	10	ND	ND	ND		ND	ND	ND	ND	ND	ND	ND	1	ND	
Vaziri et al (41)	2019	Tehran	2014-2018	606	Agar proportional	3	3	5	ND	2		ND	ND	ND	ND	ND	ND	ND	3	13	
Habibnia et al (42)	2019	Tehran	2012-2018	100	Agar proportional	15	6	ND	ND	ND		ND	ND	ND	ND	ND	ND	ND	17	ND	
Aghajani et al (43)	2019	Tehran	2011-2018	6937	Molecular	1617	1326	ND	ND	ND		ND	ND	ND	ND	ND	ND	ND	956	ND	
Amini et al (18)	2019	Tehran	2015-2017	220	Agar proportional	11	4	2	ND	ND		ND	ND	ND	ND	ND	ND	ND	11	ND	
Sakhaee et al (44)	2017	Tehran	2013-2016	395	Agar proportional	24	24	40	ND	60		ND	12	ND	ND	12	7	ND	22	4	
Khanipour et al (45)	2016	Tehran	2010-2015	723	Agar proportional	ND	ND	ND	ND	ND		ND	ND	ND	ND	ND	ND	ND	15	8	
Imani Fooladi et al (46)	2014	Tehran	2009-2011	103	Agar proportional	12	9	ND	ND	ND		ND	ND	ND	ND	ND	ND	ND	9	ND	
Tasbiti et al (47)	2017	Tehran	2006-2014	1442	Agar proportional	168	176	169	ND	330		16	13	15	13	10	12	15	33	3	
Sharifipour (48)	2014	Tehran	2011-2012	190	Agar proportional	12	5	8	ND	ND		ND	ND	ND	ND	ND	ND	ND	30	ND	
Nasiri et al (24)	2014	Tehran	2010-2012	85	Agar proportional	6	7	6	ND	14		ND	ND	ND	ND	ND	ND	ND	6	ND	
Bahrami et al (49)	2013	Tehran	2010-2012	176	Agar proportional	12	19	48	ND	ND		ND	ND	ND	ND	ND	ND	ND	10	ND	
Pooideh et al (50)	2015	Tehran	2010-2011	100	Agar proportional	13	23	26	ND	37		ND	ND	ND	61	ND	21	ND	4	ND	
Velayati et al (15)	2014	Tehran	2010-2011	324	Molecular	20	26	ND	ND	ND		ND	ND	ND	ND	ND	ND	ND	32	ND	
Varahram et al (51)	2014	Tehran	2003-2011	4825	Agar proportional Molecular	296	ND	ND	ND	ND		ND	ND	ND	ND	ND	ND	ND	ND	ND	
Moradi et al (11)	2017	West Azerbaijan	2014-2015	10	Agar proportional	0	0	ND	3	ND		ND	ND	ND	ND	ND	ND	ND	ND	ND	
Atashi et al (12)	2017	West Azerbaijan	2014	12	Agar proportional	0	1	ND	ND	ND		ND	ND	ND	ND	ND	ND	ND	ND	ND	
Sahebi et al (13)	2016	West Azerbaijan	2014	25	Molecular	1	4	ND	ND	ND		ND	ND	ND	ND	ND	ND	ND	ND	ND	
Sahebi et al (14)	2015	West Azerbaijan	2011-2013	43	Molecular	1	3	ND	ND	ND		ND	ND	ND	ND	ND	ND	ND	ND	ND	
Velayati et al (15)	2014	Yazd	2010-2011	12	Molecular	0	1	ND	ND	ND		ND	ND	ND	ND	ND	ND	ND	0	ND	
Atashi et al (12)	2017	Zanjan	2014	5	Agar proportional	0	0	ND	ND	ND		ND	ND	ND	ND	ND	ND	ND	ND	ND	

INH-isoniazid; RIF-rifampin; EMB-ethambutol; PZA-pyrazinamide; STR-streptomycin; PAS-para-aminosalicylic acid; CAP-capreomycin; CYC-cycloserine; ETO-ethionamide; OFX-ofloxacin; KAN-kanamycin; AMK-amikacin; MDR-multiple drug-resistant; XDR-extensively drug-resistant; DST-drug susceptibility testing; ND-not determined

**Table 2:** *Mycobacterium tuberculosis *antibiotic resistance profiles in different provinces of Iran during 2013-2020

Province	Antibiotic resistance (%)													
	First-line drugs				Second-line drugs									
	INH	RIF	EMB	PZA	STR	PAS	CAP	CYC	ETO	OFX	KAN	AMK	MDR	XDR
Ardabil	11.9	8.9	ND	11.1	ND	ND	ND	ND	ND	ND	ND	ND	6.1	ND
East Azerbaijan	4.3	9.9	2.4	20.1	21	ND	ND	ND	ND	ND	ND	ND	8.3	ND
Fars	16.7	16.1	7.8	ND	10.5	ND	ND	ND	ND	ND	ND	ND	13.6	ND
Golestan	3.4	1.5	0	ND	7.3	ND	ND	ND	ND	ND	ND	ND	4.2	ND
Guilan	2.5	5.1	ND	ND	ND	ND	ND	ND	ND	ND	ND	ND	7.6	ND
Hamadan	6.9	8.1	ND	40	ND	ND	ND	ND	ND	ND	ND	ND	0	ND
Hormozgan	8.2	6.1	4.1	ND	7.1	ND	ND	ND	ND	ND	ND	ND	6.7	ND
Ilam	14	8.4	ND	25	ND	ND	ND	ND	ND	ND	ND	ND	ND	ND
Isfahan	6.2	3.7	2.7	ND	4.5	ND	ND	ND	ND	ND	ND	ND	3.2	ND
Kerman	4.1	12.5	ND	ND	ND	ND	ND	ND	ND	ND	ND	ND	12.5	ND
Kermanshah	9.4	13.8	14.2	22.9	22.1	16.9	ND	3.5	12.5	ND	ND	ND	14.2	ND
Khuzestan	8.9	11.7	4	ND	36.3	ND	ND	ND	ND	ND	ND	ND	6.1	ND
Kurdistan	8.7	6.1	ND	11.1	ND	ND	ND	ND	ND	ND	ND	ND	0	ND
Lorestan	1.5	4.5	ND	21	ND	ND	ND	ND	ND	ND	ND	ND	3.5	ND
Markazi	7.4	6.3	6.9	ND	2.6	ND	ND	ND	ND	ND	ND	ND	8.6	ND
Mazandaran	3.8	5.1	ND	ND	7.4	ND	ND	ND	ND	ND	5.5	5.5	3.8	ND
Qazvin	9.4	6.1	ND	ND	ND	ND	ND	ND	ND	ND	ND	ND	20	ND
Qom	4.9	4.9	ND	ND	ND	ND	ND	ND	ND	ND	ND	ND	6.5	ND
Razavi Khorasan	7.4	7.4	5	0	4.6	ND	ND	ND	ND	ND	ND	ND	4.1	ND
Semnan	0	0	ND	ND	ND	ND	ND	ND	ND	ND	ND	ND	0	ND
Sistan and Balouchastan	4.1	4.3	0.9	ND	1.4	ND	ND	ND	ND	ND	ND	ND	1.9	ND
Tehran	7.6	7.4	7.6	ND	14.7	1.1	1.7	1	10.7	1.5	3.3	1	5.8	0.9
West Azerbaijan	3.4	10.2	ND	30	ND	ND	ND	ND	ND	ND	ND	ND	ND	ND
Yazd	0	8.3	ND	ND	ND	ND	ND	ND	ND	ND	ND	ND	0	ND
Zanjan	0	0	ND	ND	ND	ND	ND	ND	ND	ND	ND	ND	ND	ND

INH-isoniazid; RIF-rifampin; EMB-ethambutol; PZA-pyrazinamide; STR-streptomycin; PAS-para-aminosalicylic acid; CAP-capreomycin; CYC-cycloserine; ETO-ethionamide; OFX-ofloxacin; KAN-kanamycin; AMK-amikacin; MDR-multiple drug-resistant; XDR-extensively drug-resistant; ND-not determined

## Discussion

Despite the strategies initiated by the National Tuberculosis Control Program (NTP) in 1996, TB continues to be a major health concern in Iran. One of the main reasons is the emergence of drug-resistant *M. tuberculosis* strains (53). The WHO implemented a general project back in 1994 in which data on anti-TB drug resistance are collected from numerous countries; the results indicate that the prevalence of drug-resistant *M. tuberculosis* strains is still a major public health concern around the world (1). The current meta-analysis is the newest report on the *M. tuberculosis* antibiotic resistance in Iran. We believe it provides such overviews of the available data to estimate the total antibiotic resistance. Also, it helps to increase our knowledge on drug-resistant TB trends during the years to prevent further rise in drug resistance and treatment failures. Among the first-line anti-TB drugs recommended by NTP to treat new sputum-positive TB cases in Iran, isoniazid and rifampin are the most effective agents (16). Isoniazid has also been recommended as a preventive therapy in latent TB infections (54). Therefore, isoniazid- and rifampin-resistant cases can increase the risk of treatment failure due to development of MDR-TB. Our analysis showed that 6.9% and 7.9% of both new and previously treated cases between 2013 and 2020 in Iran were resistant to isoniazid and rifampin, respectively. Based on the WHO report in 2018, the global average of isoniazid resistance varies between 7.2% among new TB cases and 11.6% in previously treated TB cases (1). Additionally, findings of the present study on the prevalence of rifampin-resistant strains of *M. tuberculosis* (7.9%) is comparable with international data from the Western Pacific Region (24%), European Region (10%), South-East Asian Region (6%), African Region (3%) and Region of America (1%) (55). In 2018, WHO announced that the incidence of MDR-TB and rifampicin-resistant TB in new and previously treated TB cases were 3.4% and 18% in the world, respectively (1). The rate of MDR-TB in Iran was low (6.3%). Patients with rifampicin-resistant TB and MDR-TB must be treated with second-line drugs (1). Only a few studies have investigated *M. tuberculosis* resistance to the second-line anti-TB drugs. This could be due to a low resistance rate to first-line anti-TB drugs in Iran. In addition to MDR-TB and rifampicin-resistant TB, global surveillance and treatment of XDR-TB (defined as MDR-TB plus resistance to at least one of the fluoroquinolones and one of the injectable agents) is completely urgent (1). A total of 28 XDR-TB cases were identified in Iran and 13,068 XDR-TB cases were reported in 2018 by WHO globally (1).

Among first-line anti-TB agents, the highest and lowest resistance rates between 2013 and 2020 in Iran were seen for pyrazinamide (20.4%) and ethambutol (5.7%). It has been suggested that chromosomal mutations are associated with development of drug resistance in clinical *M. tuberculosis* strains including rifampicin resistance-associated mutations in *rpoB* gene, mutations in *katG* or *inhA* genes in isoniazid-resistant strains, *embB* or *ubiA* genes mutations in ethambutol-resistant isolates, and mutations in *pncA*, *rpsA*, *panD* and *clpC1* genes among pyrazinamide-resistant isolates (56). Similar resistance mechanisms were seen in *M. tuberculosis* isolated in Iran (data not shown). 

Furthermore, the prevalence of resistant strains to isoniazid, rifampin, ethambutol, and streptomycin as well as MDR-TB in Iran showed a downward trend from 1999 to 2020 (Figure 2). This could be attributed to differences in the methods used for assessing drug susceptibility/antibiotic resistance due to possible heterogeneity among studies (fixed- or random-effects models) or the number of included studies among two reviews. Also, decreased incidence of TB cases in Iran (21 per 100,000 population in 2011 compared with 14 cases in 2018) and fewer Afghan refugees due to sanctions, are other contributory factors. The limitations that need to be acknowledged in this study include: 1) small number of studies reporting resistance to second-line drugs and XDR-TB, 2) lack of studies reporting *M. tuberculosis* drug resistance in some provinces, 3) lack of individual separation of drug-resistant TB in many studies according to new or previously treated patients, age, ethnicity, nationality (Iranian versus Afghans), and 4) existence of heterogeneity and publication bias among included studies.

## Conclusion

The current updated systematic review and meta-analysis summarized the total prevalence of drug-resistant *M. tuberculosis* strains in new and previously treated TB cases (2013–2020) and drug-resistant TB trend (1999–2020) in Iran. Based on our results, the mean resistance to first- and second-line anti-TB drugs was low in Iran during 2013–2020 as were the cases of MDR-TB and XDR-T. There was also a decreasing trend in resistance of *M. tuberculosis* from 1999 to 2020. Hence, to continue the current downward trend and control and eliminate TB infections in Iran, continuous monitoring of resistance patterns through optimized and rapid diagnostic tests such as molecular techniques in all provinces of Iran is recommended.

## Conflicts of Interest

The author declares that there are no conflicts of interest.
